# Solvent effects on phytochemical constituent profiles and antioxidant activities, using four different extraction formulations for analysis of *Bucida buceras* L. and *Phoradendron californicum*

**DOI:** 10.1186/s13104-015-1388-1

**Published:** 2015-09-01

**Authors:** Simon B. Iloki-Assanga, Lidianys M. Lewis-Luján, Claudia L. Lara-Espinoza, Armida A. Gil-Salido, Daniela Fernandez-Angulo, Jose L. Rubio-Pino, David D. Haines

**Affiliations:** Rubio Pharma y Asociados S.A. de C.V., Blvd. García Morales, Km. 6.5 # 330. El Llano, 83210 Hermosillo, Sonora Mexico; Summative Synergy Pharmaceuticals Group (SSPG) LLC, 2040 S. Alma School Road, Suite 1, No. 255, Chandler, AZ 85286 USA

**Keywords:** DPPH, FRAP, Solvents, Phenols, Flavonoids, Phytochemical screening, *Bucida buceras* L., *Phoradendron californicum*

## Abstract

**Background:**

The present investigation evaluated 4 different solvent compositions for their relative capacity to extract total phenolic and total flavonoid (TF) components of the leaves, trunks, and stems of *Bucida buceras* L. (Combretaceae), and the stems of *Phoradendron californicum* (Viscaceae), plus mesquite and oak species endemic to the Southwestern United States, northern Mexico, and tropical regions of Central and South America, as well as to profile the composition of these plant materials and to measure their antioxidant capacity.

**Methods:**

The total phenolic content of plant material used in the present investigation was measured using the Folin–Ciocalteau assay. Total flavonoids were assayed by AlCl_3_ and 2,4-dinitrophenylhydrazin colorimetry. Nitroblue tetrazolium was utilized for scavenging of superoxide anion, and in vitro antioxidant activity was evaluated using the 2, 2-diphenyl-1-picrylhydrazyl and Ferric Reducing/Antioxidant Power assays.

**Results:**

Phytochemical screening of each plant extract evaluated revealed the following major results: (1) No evidence of alkaloids for each of the extraction phases tested was detected in the hexanic, ethanolic, or aqueous phases of *Bucida buceras* and *Phoradendron californicum* (oak and mesquite); (2) Analysis of the hexane phase of *B. buceras* and *P. californicum* (mesquite) extracts revealed the presence of carotenes, triterpenes/steroids, and lactonic groups; (3) Analysis of the ethanol and aqueous extraction phases for both plants revealed the presence of a diverse range of compounds, including tripterpenes/steroids, lactonics groups, saponins, phenols/tannins, amines and/or amino acids, and flavonoids/anthocyanins; and (4) The highest total phenolic and flavonoid content were observed in *P. californicum* (oak): 523.886 ± 51.457 µg GAE/mg extract and 409.651 ± 23.091 µg/mg of extract for methanol and aqueous fractions, respectively. The highest flavonoid content was 237.273 ± 21.250 µg PNE/mg extract in the acetone extract of *Bucida buceras* stems; while the flavonol content (260.685 ± 23.031 µg CE/mg extract) was higher in the ethanol extract of *P. californicum* (oak). The acetone extract of *B. buceras* trunk extract showed the highest levels of DPPH radical-scavenging activity (IC_50_ = 4.136 ± 0.446 µg/mL) and reducing power (4928.392 ± 281.427 µM AAE/mg extract). The highest superoxide radical scavenging activity (IC_50_) was 55.249 ± 9.829 µg/mL, observed in acetone extracts of *B. buceras* leaves.

**Conclusions:**

The results of the present investigation demonstrated the effects of extraction solvent on phenolic and flavonoid content yield—and antioxidant activities by *Bucida buceras* and *Phoradendron californicum*. The present investigation further revealed that *Bucida buceras* exhibited optimal antioxidant capacity when acetone was used as extraction solvent; and the highest yield of phenols and flavonoids were obtained from the *P. californicum* oak, using methanol and aqueous solvents, respectively.

## Background

Free radicals contribute to more than one hundred disorders in humans, including atherosclerosis, arthritis, ischemia and repercussion injury of many tissues, central nervous system injury, gastritis, and cancer [1]. Environmental pollutants, including radiation, chemicals, and dietary toxicants, along with physical trauma, cause dysregulation of immune activity, and may alter gene expression to induce expression of abnormal proteins. Oxidative processes, which trigger the production of free radicals, resulting in tissue damage, are a major contributor to diminished health, and manifested in a wide spectrum of disease [2].

Plant-derived antioxidants, especially polyphenolic compounds, have gained considerable importance due to their potential health benefits. Antioxidants are important compounds, which protect organisms from damage caused by free radical-induced oxidative stress [3]. The antioxidant activity of phenolic compounds is mainly due to their redox properties, which allow them to act as reducing agents, hydrogen donors, singlet oxygen quenchers and metal chelators [3–5]. Many plants respond to environmental stressors by producing antioxidants such as polyphenols. These absorb and neutralize free radicals, quenching singlet and triplet oxygen, or inducing expression of peroxides and other toxic metabolites [6, 7]. The medicinal value of plants is related to their phytochemical component content and secondary metabolites, including: phenolic compounds, flavonoids, alkaloids, tannins, and other stress gene response products [8, 9].

Several major groups of plant products with antioxidant and anti-inflammatory capacity have been identified. A particularly valuable class of health-enhancing plant compounds is flavonoids. These are polyphenolic molecules with properties that include free radical scavenging, inhibition of hydrolytic and oxidative enzymes, anti-inflammatory action, reduction of blood-lipids and glucose, and the enhancement of human immunity [10]. Anti-inflammatory plant metabolites are currently the focus of intensive research in the development of novel preventive and therapeutic strategies. Accordingly, methods of extracting these compounds from source material are a major focus of investigation. Current isolation and chemical purification methods used include solvent extraction processes that utilize solvent polarity as a major separation technique. These methods frequently include the use of ethyl acetate, phenol/chloroform, aqueous, and several other approaches [1, 9, 11–15].

This study examines the effects of type solvents on extraction of bioactive compounds from *Bucida buceras* L., a timber and shade evergreen tree that belongs to the family *Combretaceae*. It is endemic to the tropical regions of Northern South America, and is commonly referred to as “the nonedible black olive tree” [16]. The plants in this family are used for the treatment of various diseases in humans, including abdominal pains, chest coughs, colds, conjunctivitis, diarrhea, earache, fever, infertility in women, leprosy, pneumonia, heart diseases, sore throat, and nose bleeds [17, 18]. Diverse authors have found that *Bucida buceras* has high antifungal activity [19–21].

This investigation will also evaluate effects of the extraction of the solvent on *Phoradendron californicum* (known in United States as “dessert mistletoe” and “toji” in Mexico), which is an autotrophic hemiparasite, surviving at the expense of its higher vascular plant host [22–24]. The leaves and bark of this plant are used to treat stomachache and digestive disorders. It is also used with other plants in mixed herbal teas against venereal diseases. In a recent study, toji was found to have multiple biological activities, including anti-inflammatory and anti-proliferative effects [25]. *Bucida buceras* and *Phoradendron californicum* (mesquite and oak) are medicinal plants and both have been attributed with beneficial health effects, but there are only a few studies related to this topic. The aim of this work has been to investigate the effect of this solvent type on the yield and profile of component phenolics and flavonoids and antioxidant activity.

## Methods

### Sample sources and collection

The leaves, trunks and stems of *Bucida buceras* L. were collected in Hermosillo, Sonora, Mexico, during the summer of 2012. This genus grows in tropical areas and the tree blooms and fructifies irregularly throughout the year. The stems of *Phoradendron californicum* of *Prosopis* species (mesquite) and *Quercus* (oak) were collected from Carbo and Moctezuma towns, Sonora, Mexico, at the same year. Members of this genus grow in warm temperate and desert regions. Fruit ripening occurs in late winter to early spring. The plants were taxonomically identified by Dr. Jose Cosme Guerrero Ruiz at the Agronomy Department of Sonora University and assigned voucher specimen designations as follows: For *Bucida buceras*: RP2014-10, for *Phoradendron californicum* (mesquite): RP2014-11; and for *Phoradendron californicum* (oak): RP2014-12 deposited at the herbarium of Sonora University. All the plants were washed, dried and stored in polyethylene bags protected from light at 25 °C until analysis.

### Preparation of plant extracts

The different parts of *Bucida buceras* and *Phoradendron californicum* were dried at room temperature for 7 days, finely ground and used for extraction. The powder obtained (500 g) was mixed with 1.5 L of organic solvents, including acetone, methanol and ethanol for leaves, trunks and stems of *B. buceras*; and for the stems of *Phoradendron californicum* (mesquite and oak). All the extracts were shaken mechanically for 3 days at room temperature in an orbital shaker. The supernatant was next filtered through Whatman No. 41 filter paper by vacuum and fresh solvent was added to the samples, which were extracted for another 3 days. For aqueous extracts of *B. buceras* and *Phoradendron californicum* (mesquite and oak), 100 g of dried plants were extracted with 1L of water at 100 °C for 30 min. The residues were subsequently extracted twice with fresh aqueous solvent (water) and the extracts were combined.

Finally, the two supernatants of the different extracts of *B. buceras* and *P. californicum* were concentrated separately in a rotary evaporator (Buchi R-210, Switzerland) at 37 °C, then frozen at −77 °C and finally lyophilized at −46 °C. At last, the extracts were powdered in a coffee grinder and dissolved in dimethyl sulfoxide to a final concentration of 200 mg of dry matter/mL. All extracts were stored at −4 °C until use.

### Solvents and reagents

Catechin, rutin, pinocembrin, gallic acid, 2,2 difenil-1-picrylhydrazyl (DPPH), Folin-Ciocalteu phenol reagent, Dimetilsulfoxide (DMSO), 3,5-dinitrobenzoic acid, sodium nitrite, aluminum chloride, ascorbic acid, 2,4,6-tripyridyl-s-triazine, riboflavin, nitroblue tetrazolium and phosphate saline buffer were purchased from Sigma Chemical Co. (St. Louis, MO, USA). Sodium and pottasium hydroxide reactive grade, acetic anhydride, sodium carbonate, sodium acetate were obtained from J. T. Baker (Deventer, Holland). Absolute methanol and ethanol, acetic acid, sudan III reactive, glycerin, sodium chloride, ferric chloride, ninhydrin, chloroform, sulfuric acid, magnesium metal, amyl alcohol, iron chloride, hexane and ethylenediaminetetraacetic acid (EDTA) from Fagalab (Edo. de Mexico, Mexico), hydrochloric acid, picric acid from Fermont (Monterrey, Mexico), antimony chloride (Carr Price reactive) and Dragendorff reagent from Fluka (St. Gallen, Switzerland), fehling A and fehling B reactives from Meyer Reagents (Tlahuac, Mexico) and distilled water with mili-Q system (Millipore, Bedford, MA, USA) was used.

### Phytochemical screening

A preliminary screenings of each extract was performed following the standard phytochemical analysis protocol described by Chhabra et al. [26], with some modifications. Briefly, 10 g of the collected materials were extracted successively with hexane, ethanol and water in a Soxhlet extractor for 18–20 h. The extracts were concentrated using a rotary evaporator (Buchi R-210, Switzerland) at 37 °C and preserved at 4 °C. All extracts were subjected to qualitative chemical tests for the identification of selected phytoconstituents such as alkaloids, using Mayer´s and Draggendorff´s test, carotenes, using the Carr-Price test, triterpenes/steroids using the Liebermann-Burchard test, quinines, using the Borntrager test, lactonic groups, using the Baljet test, lipids and essential oils, using the Sudan III test, reducing compounds, using the Fehling test, saponins, using the Frothing test, phenols/tanines, using the ferric chloride test, flavonoids, using the Shinona test, amines/amino acids, using the Ninhidrin test and cardiotonic glycosides, using the Kedde test.

### Determination of total phenols and flavonoids

#### Total phenols

The total phenolic content of plant extracts was determined using the Folin-Ciocalteu reagent described by Singleton and Rossi [27] with some modification, as reported by Iloki et al. [28].

Briefly, 500 µL of plant extract samples in a range of concentrations (25–500 µg/mL) were mixed with 500 µL of Folin-Ciocalteu reagent (1:4) and 500 µL of a 10 % sodium carbonate solution (w/v) were added. The sample was left standing at room temperature (~25 °C) for 2 h and the absorbance was measured at 760 nm in a microplate spectrophotometer reader (Thermo Scientific). Based in the calibration curve established as preparation for the experiments, (0.00625–0.1 mg/mL), the results were expressed as micrograms of gallic acid equivalent per milligram of extract (µg GAE/mg).

#### Flavonoids

The total flavonoid content of each batch of plant material was measured by two complementary colorimetric methods, one by the method aluminum chloride (AlCl_3_) for the quantification of flavones and flavonols that react better with AlCl_3_ and other by the method of 2,4-dinitrophenylhydrazin (DNP) for flavanones and flavanonols [29, 30].

### Aluminum chloride (AlCl_3_) method

The colorimetric aluminum chloride method was used to determine flavonoid content according to the method proposed by Zou et al. [31]. The method is based on quantification of the yellow-orange color produced by the interaction of flavonoid with AlCl_3_. A 50 μL aliquot of plant extract appropriately diluted, was mixed with 1250 μL of deionized water and 75 μL of 5 % sodium nitrite, after 6 min, 150 μL of 10 % AlCl_3_ solution was added and the mixture was allowed to stand for 5 min; followed by addition of 500 μL of 1 M sodium hydroxide. After 30 min of reaction, absorbance was read at 510 nm. The flavonoid content was assessed by reference to a calibration curve of catechin (0.2–1.0 mg/mL) and expressed as μg of catechin equivalent per mg of extract (μg CE/mg).

#### 2,4-Dinitrophenylhidrazin (DNP) method

Quantification of flavanones and flavanonols was accomplished using a spectrophotometric method proposed by Nagy and Grancai [32], which was based on the interaction of these compounds with DNP in acid medium, to form phenylhydrazones. 40 µL of sample were dissolved in 80 µL of DNP solution (For 5 mL: 50 mg of DNP in 100 µL of 96 % sulfuric acid and 4850 µL of methanol), mixed and incubated at 50 °C for 50 min in a water bath, cooled at room temperature and 280 µL of 10 % potassium hydroxide (KOH) in water were added. Finally, the absorbance was measured at 486 nm. The total content of flavanones was determined using a calibration curve based on pinocembrin (0.5–5.0 mg/mL) and expressed as μg of pinocembrin equivalent per milligram of extract (μg PNE/mg).

### Antioxidant activity

#### Free-radical scavenging activity (DPPH assay)

The free-radical scavenging activity of each plant material was measured as described previously with some modifications [33]. Briefly, 500 µL of (0.2 mM) 1,1-diphenyl-2-picrylhydrazyl (DPPH) in ethanol was added to 500 µL of selected concentrations (12–100 µg/mL) of the extracts and the mixture was incubated for 30 min in the dark, at room temperature. Absorbance of the mixture was measured using a microplate spectrophotometer reader (Thermo Scientific) at 517 nm. The assay vehicle without extract, served as an absolute control (A blank). The free radical scavenging of DPPH was calculated according to the following formula:

$$\% {\text{ inhibition}} = \left( {{\text{A}}_{\text{blank}} - {\text{A}}_{\text{sample}} /{\text{A}}_{\text{blank}} } \right) \times { 1}00$$

where A_blank_ is the absorbance of the control reaction (containing DPPH solution adequately diluted with ethanol) and A_sample_ is the absorbance of the test compound. Extract concentrations mediating 50 % inhibition (IC_50_) was calculated from a graphic plot of inhibition percentage versus extract concentration. The lower the IC_50_, the higher the antioxidant activity of the examined compound.

#### Ferric reducing antioxidant power assay (FRAP assay)

The FRAP reagent was prepared in acetate buffer (pH 3.6), 10 mmol 2,4,6-tripyridyl-s-triazine (TPTZ) solution in 40 mmol hydrochlorin acid and 20 mmol iron (III) chloride solution in proportions of 10:1:1 (v/v), respectively. The FRAP reagent was prepared daily. 5 µL of samples at 0.5 to 2 mg/mL diluted with 20 μL of distilled water were added to 150 μL of FRAP reagent [34]. The absorbance of the mixture was measured using microplate spectrophotometer reader Thermo Scientific at 593 nm after 8 min. The standard curve was prepared with ascorbic acid (AA) and the results were expressed as μmol AA Equivalent/mg polyphenol-rich extract.

### Superoxide radical scavenging

Superoxide anion radical scavenging activity was determined by the method proposed by Beauchamp and Fridovich [35] with little modifications. The assay is based on the capacity of the sample to inhibit formazan blue formation by scavenging the superoxide radicals generated in the riboflavin-light-nitroblue tetrazolium (NBT) system. The reaction medium contains 625 µL phosphate buffer (pH 7.6), 25 µL riboflavin (0.2 mg/mL), 50 µL EDTA (12 mM), 25 µL NBT (1 mg/mL) and 25 µL of different concentration of sample. Illuminating the reaction mixture for 10 min started the reaction. Superoxide radical content of each sample was measured spectrophotometrically by the increase in the amount of the absorbance at 590 nm. Blank standardization was performed in the same way with 25 µL of ethanol in the place of samples being of tested. The concentration of test sample required to inhibit NBT reduction by 50 % (IC_50_) was calculated from dose-inhibition curves.

### Statistical analysis

All data were expressed as mean ± standard deviation. Statistical analysis was performed by analysis of variance (ANOVA). A post hoc test (Turkey) was carried out when the differences shown by data were significant (p < 0.05). NCSS (version 2007) statistical program was used for all analysis included the Pearson’s correlation coefficient.

## Results and discussion

### Phytochemical screening

Preliminary phytochemical screening of *Bucida buceras* L. and *Phoradendron californicum* extract show the presence of various bioactive components which are shown in Table 1. The results provide evidence of the presence of carotenes, triterpenes/steroids, lactonic groups, phenols/tannins, amines or amino acids, flavonoids/anthocyanins, saponins, reducing compounds and lipids/essential oils in selected extracts of *B. buceras* and *P. californicum* (mesquite and oak), except the last group for *B. buceras*. The results also revealed the absence of alkaloids and quinines with the exception of *P. californicum* (mesquite) on the ethanolic phase in which the presence of quinines was detected.

**Table 1:** Results of phytochemical screening for the different plant extracts

Phase	Secondary metabolite	Plant extracts		
		*B. buceras*	*P. californicum* mesquite	*P. californicum* Oak
Hexanic	Alkaloids	–	–	–
	Carotenes	+++	+++	+
	Tripterpenes/steroids	+++	++	+
	Quinines	–	–	–
	Lactonic groups	+++	+++	+
	Lipids and/or essential oils	–	+	+
Ethanolic	Alkaloids	–	–	–
	Tripterpenes/steroids	++	++	+++
	Quinines	–	++	–
	Lactonic groups	+++	++	+
	Reducing compounds	–	++	++
	Saponins	+++	++	+
	Phenols/tannins	+++	+++	+++
	Amines and/or amino acids	+	+++	+++
	Flavonoids/anthocyanins	++	++	+
	Cardiac glycosides	+	–	–
Aqueous	Alkaloids	–	–	–
	Saponins	++	++	+++
	Phenols/tannins	+++	+++	+++
	Reducing compounds	+	++	++
	Flavonoids/anthocyanins	+	+++	+++

–, absent; +, low in abundance; ++, moderate in abundance; +++, high in abundance

Some authors have found the presence of diterpenes and flavanones (flavonoids) in *Bucida buceras* [36, 37], this result agree with some of the results obtained in this investigation. Meanwhile, Iloki et al. [33] found that *P. californicum* is a plant rich in flavonoids, saponins, phenols and/or tannins mainly.

Major outcomes of the present investigation revealed that the samples tested contained high concentrations of health-enhancing phytochemical constituents, including flavonoids, phenols and/or tannins, saponins, amines and/or amino acids [25]. Plants endemic to Northwest Mexico such as *Phoradendron californicum* contained compounds with significant antioxidant and antiproliferative activities. Hayashi et al. [36, 37] identified presence of potent cytotoxic agents including Bucidarasins and Buceracidins in *Bucida buceras*. Moreover, many flavonoids and terpenoids are potent antioxidants, with anti-inflammatory, antibacterial, antiviral and anticancer properties [38, 39]. Other compounds previously identified in these plant materials include phenolics, which possess antioxidative, antidiabetic, anticarcinogenic, antimicrobial, antiallergic, anti-inflammatory and antimutagenic activities [40, 41]. These plants also contain steroids, which are known to mediate cardiotonic activities and possess insecticidal and antimicrobial properties, while tannins, which are also found in these plants, are known to possess general antimicrobial and antioxidant activities [42]. The saponins fraction of extracts described here, are typically used in treatment of hypercholesterolemia, hyperglycemia, as antioxidants, anticancer, antifungal, antibacterial, anti-inflammatory and in weight loss [40, 43].

### Total phenolic and flavonoid content determination

#### Total phenolic content (TPC)

**Fig. 1:**
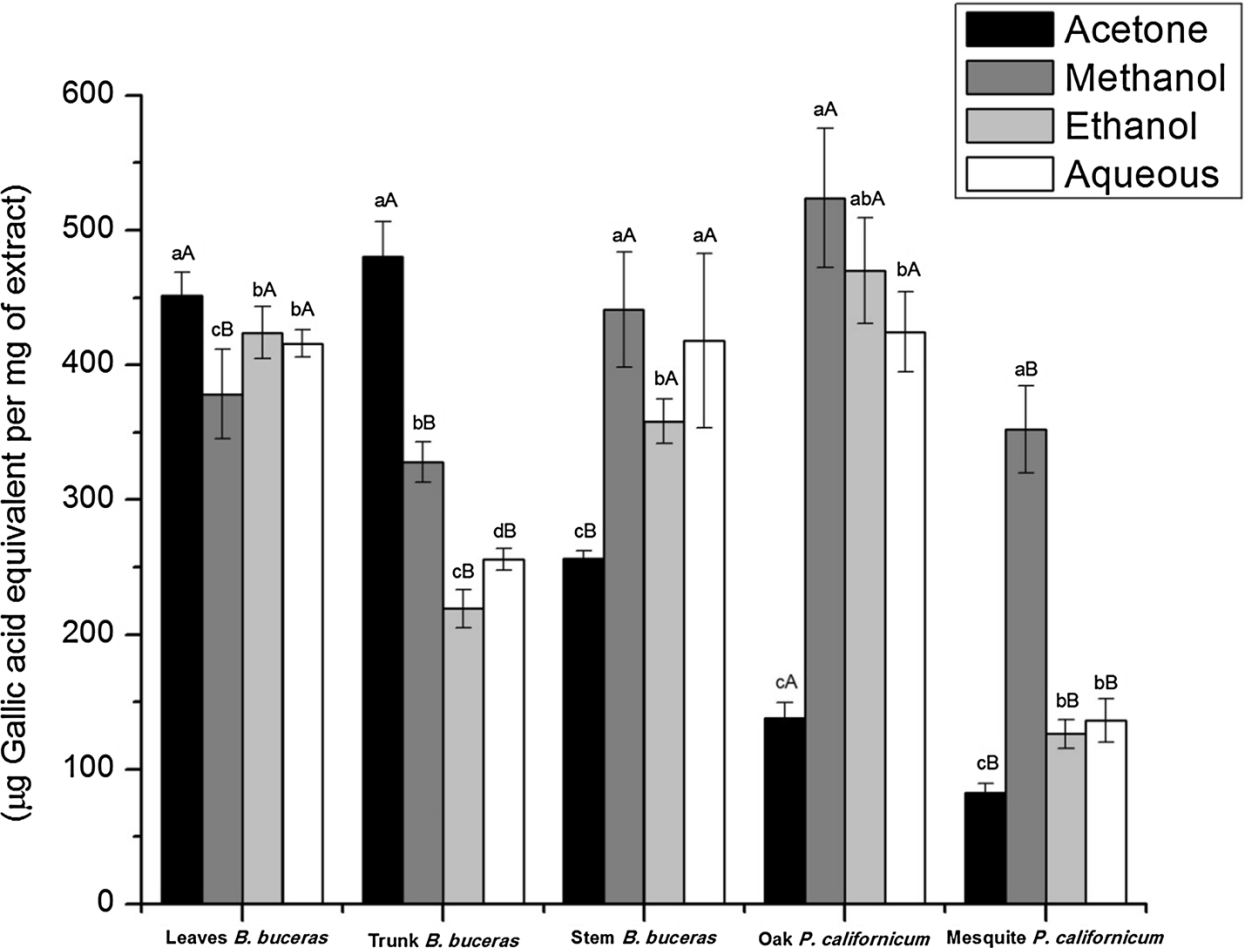
Total phenols content from *B. buceras* and *P. californicum* of oak and mesquite in different solvent type. Values as mean ± standard deviation (n = 3) × triplicate. *Different lowercase letter* indicate significant differences (p < 0.05) between different solvent type in the same plant part and *different capital letter* indicate significant differences (p < 0.05) between the same solvent type in different plant part for *B. buceras* and same solvent type in same part (stems) for *P. californicum* (oak and mesquite)

Sample content of phenolic compounds (µg GAE/mg of extract) in different solvent extracts of the leaves, stems and trunks of *B. buceras* L. and stems of *P. californicum* of mesquite and oak are summarized in Fig. 1.

Results of the assays for phenolics described in the present report, indicated a wide variation in the total phenolic content in the different extracts, ranging from 82.818 to 523.886 µg GAE/mg of extract, showing the higher phenolic content the methanol, ethanol Oak toji extracts and acetone trunk and leaves of *B. buceras* extract.

Results of these assays, demonstrated significant variability in total yield of phenolic compounds (p < 0.05). In the present study, methanol proved to be the most effective solvent for isolation of phenolic compounds (327.91–523.89 µg GAE/mg of extract) from samples of *B. buceras* and *P. californicum*, whereas much lower yields were obtained from samples extracted with acetone (82.82–480.11 µg GAE/mg). The order of effectiveness in extraction of phenolics was methanol (388.04 µg GAE/mg) >aqueous (321.05 µg GAE/mg) ≥ethanol (304.34 µg GAE/mg) ≥acetone (283.49 µg GAE/mg). In general, extractability of a particular component appeared to depend on extraction medium polarity and the ratio of solute to solvent. Moreover, recovery of phenolic compounds appeared dependent on the type of solvent used, its polarity index (PI) and the solubility of phenolic compounds in the extraction solvents.

The solubility of polyphenols was observed to depend mainly on the presence and position of hydroxyl groups and the molecular size and the length of constituent hydrocarbon chains. Phenolic compounds are often extracted in higher amounts in more polar solvents. In such cases aqueous extract (PI = 9) was not more efficient in extracting polyphenolic compounds than methanol (PI = 6.6), a phenomenon that may be due at least in part, to the thermal decomposition of some phenolic antioxidants at the higher temperatures which were used for this extraction. This effect was evident, despite the observation that increased heating temperature may increase the yield of phenols in an extraction. Results shown here demonstrate that solvents with similar polarity ethanol (PI = 5.2) and acetone (PI = 5.4) have significantly different effects on polyphenol content, mainly for trunk and stem of *B. buceras* and Toji Oak. These significant variations indicated that change in phenolic solubility may be altered dependent on particular extraction conditions.

Whereas certain compounds are common to most parts of a particular plant, none are ubiquitously abundant and most are restricted in distribution to a defined location of plant anatomy (e.g. seeds, stems, leaves, bark, etc.). The present study revealed that the distribution of polyphenols differed significantly between different of *B. buceras* parts for all solvents. For example, leaf content of these compounds was 417.41 µg GAE/mg of extract; and stems contained 355.53 µg GAE/mg of extract which were approximately 15 and 30 % more than the phenolic content of the trunks (314.24 µg GAE/mg). This difference was particularly marked in ethanol and aqueous solvents. Significant differences were found between extraction yields of phenolics, dependent on types of solvent used; and origin of a sample on plant anatomy. It was observed that the highest extraction yield of phenolic compounds was achieved in acetone for *B. buceras* leaves and trunk.

Significant solvent-dependent differences in content of phenolic compounds were observed when samples from *P. californicum* (oak and mesquite) were evaluated. Contributing factors may be related to environments in which the plants lived. Oaks from highland forests are adapted to cold and acid soils. Environmental stressors to which the plants are subjected favor expression of stress-response compounds, including polyphenols. In the present study, methanolic extraction protocols were the most efficient for recovery of phenols.

Different parts of the same plant may synthesize and accumulate different compounds or different amounts of a particular compound due to their differential gene expression, which in turn, affects antioxidant activities and other biological properties of the plant extracts [44, 45]. Many studies have confirmed that the amount and composition of phenolic and flavonoid compounds is diversified at the sub-cellular level and within plant tissues as well [46, 47].

In general, the results of the study revealed that methanol extracted higher levels of phenolics from Toji Oak than the other solvent systems tested. These results were similar to those reported for Toji Oak by Iloki et al. [33]. In these investigations, methanolic extracts of Toji Oak and Mesquite contained the highest total amounts of phenolics, flavonoids and condensed tannin compounds, when compared to extraction yields of the other solvent systems: (acetone, methanol and water). Further, results shown here are similar to those reported by Sun and Ho [48] where methanol solvent was most effective in extracting phenolic components from oat bran. Methanol and ethanol have been proven as effective solvents for extraction of phenolic compounds [14]. Conversely, Jayaprakasha et al. [49] demonstrated that acetone, methanol and water were relatively ineffective for the extraction of total phenols from grapes seeds.

The recovery of phenolic contents in different samples is influenced by the polarity of extracting solvents and the solubility of each compound in the solvent used for the extraction process [50, 51]. Therefore, it is difficult to select an optimally appropriate solvent for extraction of phenolics from multiple plant material samples [52]. The profile and yield of polyphenol content and antioxidant activity appears higher in more polar solvents [16], hence these seem good choices for screening of multi-sample substances.

In the context of these observations, it should be noted that the phenolic compounds are often associated with other biomolecules (polysaccharides, proteins, terpenes, chlorophyll, inorganic compounds) and a solvent suitable for the extraction of particular classes of compound must be used based on the structural features and related level of aqueous solubility of a particular target molecule [9]. Using this general strategy, the phenolic extraction efficiencies of the different parts of Bucida and Toji may be optimized, based on the relative polarities of target compounds. Thus different classes of solvent may therefore be required depending on the known distribution of target compounds in various plant anatomical locations.

#### Total flavonoid content (TFC)

Total flavonoid content was evaluated in the present study using two colorimetric methods. The flavonol/flavones content in the different extracts of plant materials was evaluated using catechin equivalent as a reference; and the flavanones/flavanonols content was evaluated in pinocembrin equivalent as a reference. Some insight into the molecular mechanisms contributing to solvent extraction efficiency may be gained by considering major features of target compounds. For example, the aluminum chloride method involves formation of stable acid complexes between the AlCl_3_ reagent and the C-4 keto group; and either the C-3 or C-5 hydroxyl group of flavones and flavonols. In addition, aluminum chloride forms labile acid complexes with the ortho-dihydroxyl groups in the A- or B-ring of flavonoids.

The 2, 4-dinitrophenyl-hydrazine reagent, (DNP) reacts with ketones and aldehydes to form 2,4 dinitrophenylhydrazones, although flavones, flavonols and isoflavones with the C2-C3 double bond fail to react with 2,4-dinitrophenyl-hydrazine and only flavanones strongly react.

Of the plant materials studied here, the highest yields of flavanones and flavanonols were found in *Bucida buceras*. In trunk and stem samples, flavanones yields were 2–3 times greater than those of flavones/flavonols (Table 2). The flavanone content in the trunk was greater than the leaves and stem, except in acetone extracts of the stem. Non-significant differences between solvents were observed in analysis of phenolic content of trunks, whereas for leaves and stems, ethanol and acetone contained the highest flavanone content. These findings suggest that acetone extraction appears superior to other solvents for recovery of flavonols/flavones and flavanones (total flavonoids) from *Bucida buceras*.

**Table 2:** Flavonoids content by the two colorimetric methods on *B.* *buceras* and *P*. *californicum* in different solvent type

Plant	Part	Solvent	Flavonols (µg of CE/mg of extract)	Flavanones (µg of PNE/mg of extract)	Total flavonoids (µg/mg of extract)
Bucida	Leaf	AC	^a^72.075 ± 8.791^A^	^b^69.897 ± 4.481^C^	^a^142.591 ± 13.001^B^
		ET	^b^37.219 ± 9.716^A^	^a^95.837 ± 6.389^A^	^ab^133.056 ± 12.590^A^
		ME	^a^71.183 ± 11.033^A^	^c^54.475 ± 6.355^B^	^ab^90.067 ± 33.427^AB^
		A	^b^51.704 ± 9.873^A^	^b^67.922 ± 10.195^B^	^b^120.785 ± 7.593^A^
Bucida	Trunk	AC	^a^51.180 ± 6.242^B^	^a^119.538 ± 11.551^B^	^a^169.034 ± 16.231^B^
		ET	^d^19.119 ± 2.018^B^	^a^93.999 ± 8.614^A^	^c^112.710 ± 9.607^A^
		ME	^c^33.976 ± 5.117^B^	^a^104.269 ± 15.674^A^	^b^138.246 ± 15.296^A^
		A	^b^40.306 ± 2.892^A^	^a^107.886 ± 14.794^A^	^ab^148.947 ± 16.008^A^
Bucida	Stem	AC	^a^49.980 ± 6.171^B^	^a^237.273 ± 21.254^A^	^a^287.254 ± 20.597^A^
		ET	^c^24.643 ± 2.957^A^	^b^49.013 ± 3.959^B^	^c^73.656 ± 5.214^B^
		ME	^b^34.875 ± 3.671^B^	^b^64.122 ± 5.262^B^	^b^98.997 ± 6.601^B^
		A	^b^41.430 ± 2.510^A^	^b^62.979 ± 5.292^B^	^b^104.409 ± 4.293^A^
Toji mesquite	Stem	AC	^d^25.811 ± 3.523^B^	^a^42.977 ± 4.286^B^	^d^68.788 ± 6.022^B^
		ET	^c^68.094 ± 7.528^B^	^a^42.987 ± 4.897^A^	^c^114.867 ± 10.811^B^
		ME	^a^153.673 ± 11.553^A^	^b^34.542 ± 3.395^B^	^a^190.125 ± 8.410^B^
		A	^b^90.516 ± 7.289^B^	^a^49.45671 ± 4.190^B^	^b^138.100 ± 8.310^B^
Toji oak	Stem	AC	^d^87.632 ± 6.974^A^	^b^63.414 ± 3.651^A^	^d^151.097 ± 9.766^A^
		ET	^b^260.685 ± 23.031^A^	^b^54.719 ± 5.671^A^	^b^330.295 ± 15.120^A^
		ME	^c^173.377 ± 14.982^A^	^b^59.170 ± 4.895^A^	^c^233.384 ± 14.325^A^
		A	^a^316.858 ± 13.580^A^	^a^83.023 ± 11.668^A^	^a^409.651 ± 23.091^A^

The results are expressed as mean ± standard deviation (n = 3) of triplicate samples. Values in the same column followed by a different lowercase letter are significantly different (p < 0.05) in the same plant and part with different solvent. Different capital letter indicate significant differences (p < 0.05) between different part of the plant in the same solvent for *B. buceras* and same part and solvent for *P. californicum* (oak and mesquite)

*ET* ethanol, *A* aqueous, *AC* acetone, *ME* methanol, *CE* Catechin, *PNE* Pinocembrine

Assays conducted on *Phoradendron californicum* revealed that the content of flavonols/flavones was approximately 3-fold higher than that of flavanones. Analysis of plant materials showed that Toji (Oak) contained the highest flavonol/flavone content compared to toji (Mesquite). In contrast to *Bucida buceras*, aqueous and methanol extracts exhibited the highest content of phenolic compounds; while the acetone solvent was less effective in extracting flavonols/flavones components from Toji (oak and mesquite). These outcomes indicate that polyphenols from *Phoradendron californicum* were extracted most efficiently in more polar solvents.

The amount of total flavonoids extracted from *Bucida buceras* and *Phoradendron californicum* using different solvents type ranged from 68.788 ± 6.022 to 409.651 ± 23.091 µg/mg of extract, with the highest amount of total flavonoids observed in the aqueous extract of oak, whereas the lowest yield was obtained in the acetone extract of mesquite.

Results of the present study demonstrated higher total flavonoid content in *Phoradendron californicum* (mesquite) than was previously reported by Jiménez et al. [25]. It has been established that phenolics are the major plant compounds with antioxidant activity, a property derived from their redox abilities. Phenolic compounds are a class of antioxidant agents, which can quench and neutralize the free radicals [53]. Flavonoids and flavonols are two other major classes of health-enhancing plant compounds and are among the most widely distributed groups of compound found in the plants with known benefit to vertebrate viability. Both flavonoids and flavonols possess antioxidant activity as a result of a native scavenging or chelating attribute [54].

### Evaluation of the antioxidant activity

#### DPPH radical scavenging assay

**Fig. 2:**
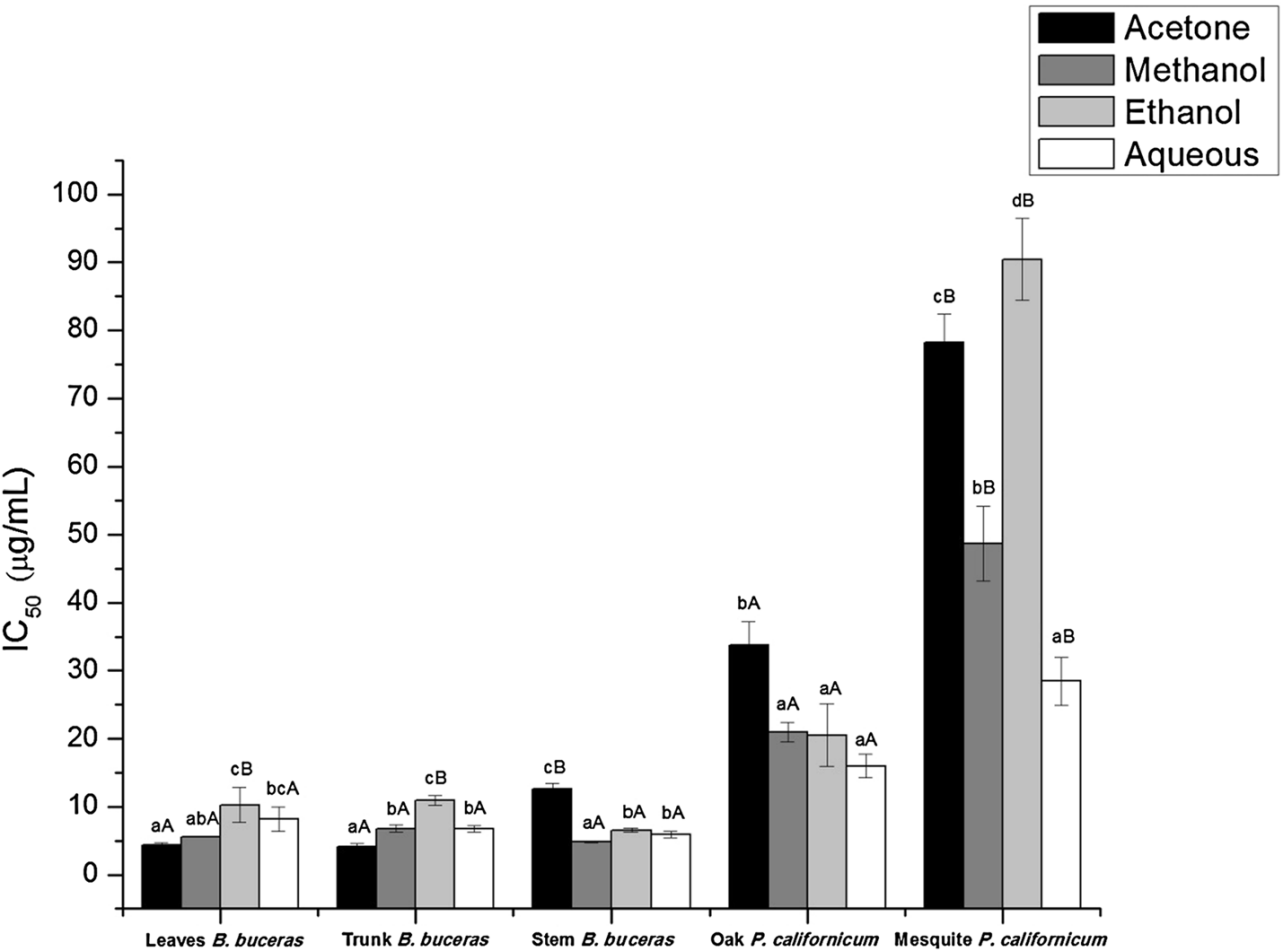
DPPH radical scavenging activity of the different extracts from *B. buceras* and *P. californicum* of mesquite and oak. Values as mean ± standard deviation (n = 3) × triplicate. *Different lowercase letter* indicate significant differences (p < 0.05) between different solvent type in the same plant part and *different capital letter* indicate significant differences (p < 0.05) between the same solvent type in different plant part for *B. buceras* and same solvent type in same part (stems) for *P. californicum* (oak and mesquite)

DPPH radical scavenging activity of the various *Bucida buceras* and *Phoradendron californicum* (mesquite and oak) extracts evaluated in the present investigation are shown in Fig. 2. The extracts of each plant examined in the present study, exhibited free radical scavenging properties and low IC_50_ values, which are indicative of robust antioxidant capacities. The IC_50_ values for free radical scavenging activity were observed to be in a range of 4.136 ± 0.446–90.265 ± 6.032 µg/mL. Maximum scavenging activity in the samples studied, was observed in by *Bucida buceras* (4.13–12.79 µg/mL), followed by *Phoradendron californicum*-oak (16.03–33.73 µg/mL) and *Phoradendron californicum*-mesquite (28.45–90.265 µg/mL).

For the purpose of the present investigation, acetone extracts of leaves and trunk of *Bucida buceras* exhibited potent scavenging capacity against the free radical DPPH. Moreover, with this same solvent (acetone), the highest content of polyphenols was observed. Significant differences (p < 0.05) in free radical scavenging capacity between extracts using the different solvent were observed. In decreasing order of scavenging effectiveness, this included: acetone > methanol ≈ aqueous > ethanol (Fig. 2). These outcomes demonstrated that high-polarity solvents (aqueous and methanol) were less effective for extraction of antioxidants with efficient free radical scavenging properties versus an intermediate-polarity solvent (acetone) but not ethanol. Acetone extracts of *Bucida buceras* trunk showed the highest DPPH radical-scavenging activity (4.136 ± 0.446 µg/mL), which can be attributed to their high phenolic contents. These results were much better than those obtained in an acetone extract of trunks of *Delonix regia* (44.64 ± 1.8 µg/mL) conducted by Shabir et al. [55]. Meanwhile, Masoko and Eloff [56] found a strong antioxidant activity in different plants of the family Combretaceae in acetone solvent. Also, this value was higher than the values of standards like ascorbic acid and rutin (10.0 and 29.0 µg/mL respectively) [57] showing high scavenging of DPPH radical.

Significant influence of the polarity solvent on antioxidant activity was observed for different parts of *Bucida buceras* plant anatomy. While the DPPH scavenging activity was not significantly different when comparisons were made between leaves, trunk and stem in high polarity solvents (water and methanol), significant differences emerged when moderately polar solvents (acetone and ethanol) were used to extract *Bucida buceras* stem samples. Sultana et al. [15] found that the ethanolic extract of *Terminalia arjuna* showed the highest scavenging activity. Contrary, Jegadeesware et al. [58] found the lowest IC_50_ value in ethyl acetate, a solvent much less polar (PI = 4.4).

Conversely, methanol, ethanol and aqueous extracts from *Phoradendron californicum*-oak exhibited higher total phenolic and flavonol/flavone content than *Bucida buceras* extracts, but not antioxidant activity. It is possible in this context phenolic compounds (flavanones) produced by *Bucida buceras* possess an ideal structure for the scavenging of free radicals since structure of these molecules includes a number of hydroxyls acting as hydrogen donators which makes them very powerful antioxidant agents. The higher efficiency of free radical inhibition of the *Bucida buceras* extracts can be related to its higher flavanone content, rather than to flavonols/flavones, which are predominant in the extract. In addition, a moderate and significant correlation (r = −0.52, p < 0.001) between total phenolic and free radical scavenging was observed for this plant. Also the high abundance of carotene detected by phytochemical screening of *Bucida buceras* may account for this activity.

Among the variants of *Phoradendron californicum*, toji-Oak exhibited 2–4 times greater antioxidant activity than toji-Mesquite. The observed ratio of oak-mesquite activity was more variable: approximately 2:1 in the aqueous and methanol extracts, 3:1 in the acetone and 4.5:1 in the ethanol extract. Results of the present study reveal high and significant correlations between the content of total phenolic (r = −0.78, p < 0.001) and flavonols/flavones (r = −0.73, p < 0.001) with the antiradical activity of *Phoradendron californicum*-oak. Similar results were reported on *Zingiber officinaleRoscoe* (ginger). DPPH activities vary with high levels of total phenols and total flavonoids and exhibit high free radical scavenging activity [59, 60].

Previous investigations have demonstrated that alteration in solvent polarity may be used to differentially precipitate selected antioxidant compounds [1, 61]. In the present study, significant differences in free radical scavenging ability for extracts of *Phoradendron californicum*-mesquite were observed dependent on the kind of extraction solvent used, while in *Phoradendron californicum*-oak the effect of solvent was minimized. Aqueous and methanol extracts showed the strongest scavenging ability, followed by acetone and ethanol extract.

#### Ferric reducing antioxidant power assay (FRAP assay)

**Fig. 3:**
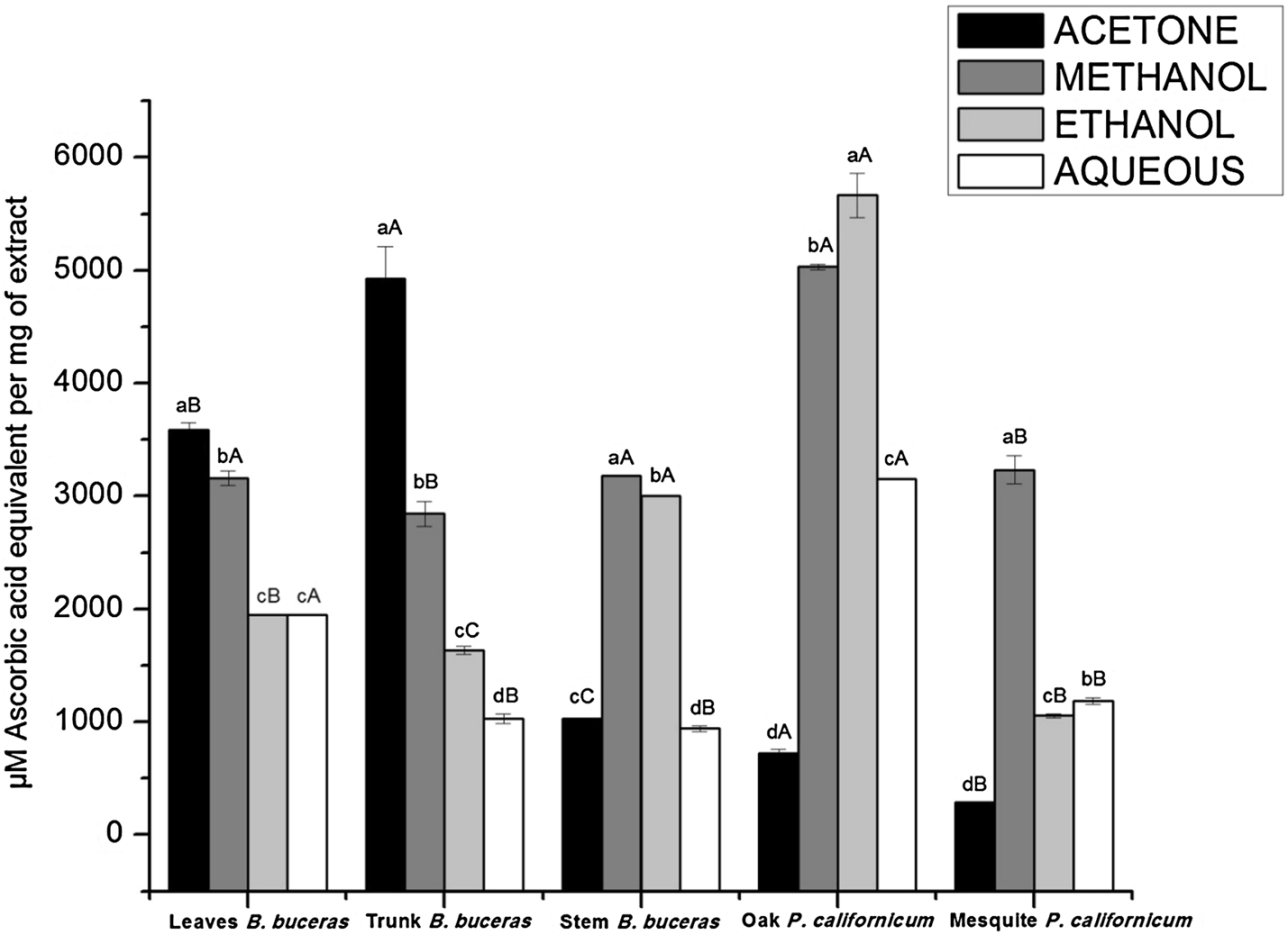
Reducing power of different extracts from *B. buceras* and *P. californicum* of mesquite and oak. Values as mean ± standard deviation (n = 3) × triplicate. *Different lowercase letter* indicate significant differences (p < 0.05) between different solvent type in the same plant part and *different capital letter* indicate significant differences (p < 0.05) between the same solvent type in different plant part for *B. buceras* and same solvent type in same part (stems) for *P. californicum* (oak and mesquite)

The reducing power of the plant extracts ranged from 283.899 ± 3.912 to 4928 ± 281.427 µM AAE/mg of extract. Figure 3 shows the ferric reducing antioxidant power of the different extracts of *B. buceras* and *P. californicum* (oak and mesquite). The highest reducing power was exhibited by the acetone trunk *Bucida buceras* wish is also high in phenolic content (480.113 ± 26.044 µg GAE/mg of extract) and the acetone *P. californicum* (mesquite) showed lowest activity. Diverse studies have shown that acetone or its combinations with water show better reducing antioxidant power and partially accord with the results obtained in this investigation [52, 62, 63].

In this assay, the presence of antioxidants in the extracts reduced Fe^+3^/TPTZ complex to the ferrous form. The intensity of the blue color formation is proportional to the concentration of the ferrous form and the antioxidant capacity of the extract. Antioxidant compounds that exhibit antioxidant capacity in FRAP assay are usually electron donors. FRAP assay had a similar trend to DPPH radical assay. A significant correlation was found between IC_50_ values of DPPH and FRAP (r = −0.69, p < 0.001). The reducing capacity of the extracts may serve as an indicator of potential antioxidant activities through the action of breaking the free radical chain by donating hydrogen atom [64].

Results shown here indicate that methanol solvent exhibits comparatively high reducing power for different parts of *B. buceras* and type of *P. californicum* due to the high polarity of the solvent system (Fig. 3). These results agree with the results obtained by Barchan et al. [65] were the methanolic extract of *Mentha* tree showed high reducing power. The reducing potential of antioxidant components is closely associated with their total phenolic content. The extracts with higher levels of total phenolics, also exhibit greater ferric reducing power. In the aforementioned examples (*B. buceras* and type of *P. californicum*), the correlation between FRAP with phenolic and flavonoid contents was 0.806 (p < 0.001) and 0.590 (p < 0.001) respectively. In *B. buceras* among different parts, trunk and leaves exhibited the highest ferric reducing antioxidant power with respect to stem samples—an effect that might be due to the greater polyphenol content in these parts. *P. californicum*-oak exhibited higher reducing power than *P. californicum*-mesquite mainly in aqueous and ethanolic extracts.

#### Superoxide radical scavenging activity (O_2-)_

Superoxide radical is a potent reactive oxygen species and is highly toxic at fairly low tissue concentrations [66]. Moreover, although this anion is a weak oxidant, it gives reacts with other molecules in tissues to form highly toxic hydroxyl radicals as well as single oxygen, both of which are significant contributors to oxidative stress [67].

**Fig. 4:**
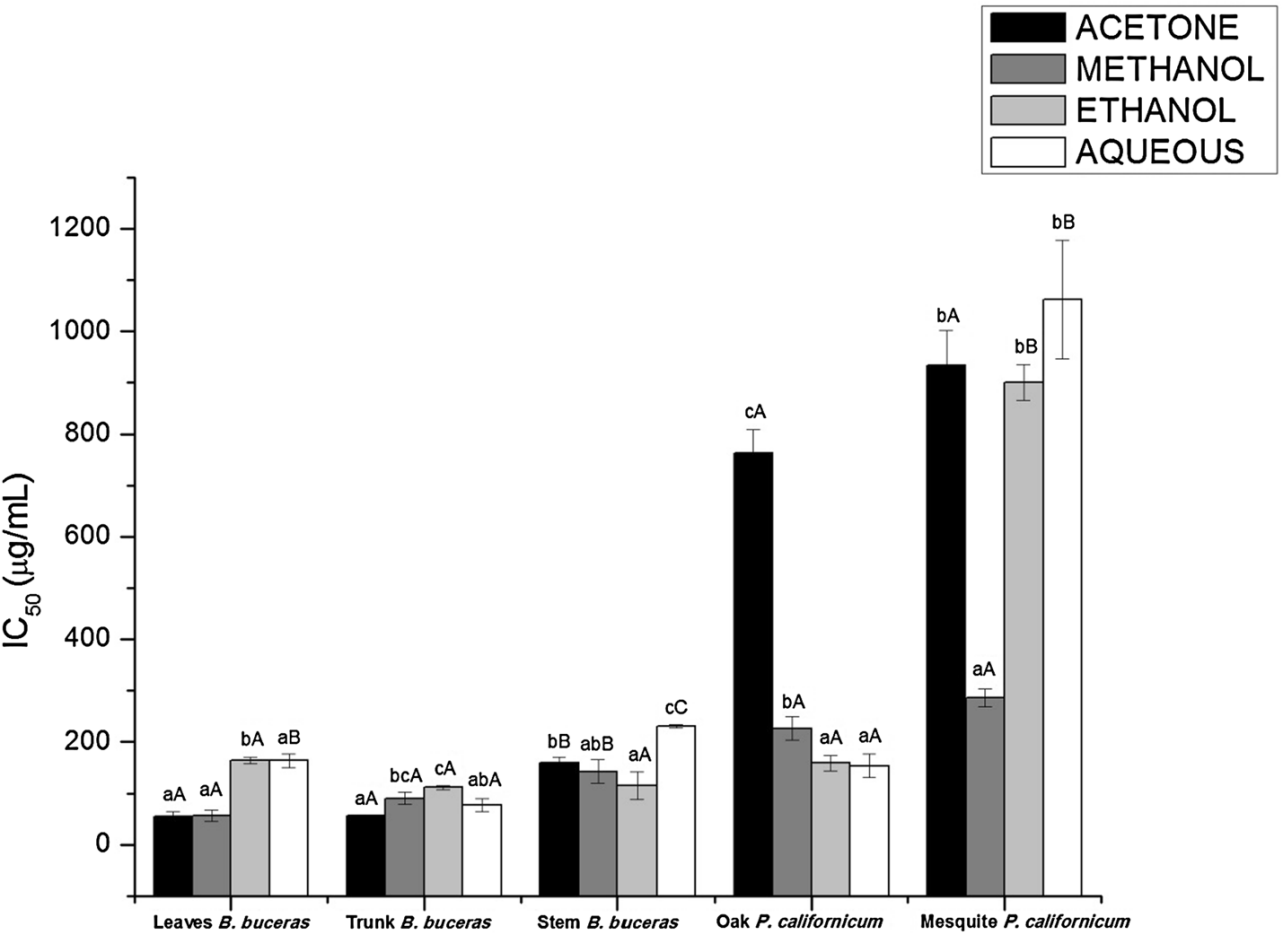
Superoxide radical scavenging activity of the different extracts from *B. buceras* and of mesquite and oak. Values as mean ± standard deviation (n = 3) × triplicate. *Different lowercase letter* indicate significant differences (p < 0.05) between different solvent type in the same plant part and *different capital letter* indicate significant differences (p < 0.05) between the same solvent type in different plant part for *B. buceras* and same solvent type in same part (stems) for *P. californicum* (oak and mesquite)

Results of O_2-_ activity assays conducted in this study, yielded, values ranging between 55.249 ± 9.829 and 1062.17 ± 116.172 µg/mL. Figure 4 shows the results of assays for superoxide radical scavenging activity of the different extracts of *Bucida buceras* and *Phoradendron californicum* (mesquite and oak). The acetone leaves and trunk *Bucida buceras* showed the best superoxide radical scavenging activity and the aqueous *P. californicum* gave the lowest.

The low IC_50_ values in *Bucida buceras* followed by *Phoradendron californicum* oak show a high capacity for superoxide scavenging of these extracts while *Phoradendron californicum*-mesquite exhibited a fivefold less activity. Similar results were observed in assays for the ability to scavenge the DPPH radical and significant association was observed between these antioxidant activities (r = 0.7444, p < 0.001). Methanolic extracts exhibited the most robust superoxide scavenging ability for phenolic compounds (r = −0.760, p < 0.001). Extracts of trunk and leaves from *Bucida buceras* were found to possess a strong and potent superoxide scavenging capacity with respect to analyses for these parameters in stems. The scavenging activities of leaves and trunk of *Bucida buceras* were similar to that of standard reference compounds such as scopoletin (54.5 µg/mL), quercetin (70.3 µg/mL) and catechin (73.3 µg/mL).

## Conclusions

The present study revealed the presence of different phytochemicals such as phenolic compounds, carotenes, saponines, aminoacids among others; and the absence of alkaloids and quinines in the plant materials analyzed. The in vitro antioxidant activity (DPPH, FRAP, O_2_-) and content of phenolic compounds for four solvent extracts of *Bucida buceras* and *Phoradendron californicum* (mesquite and oak) were evaluated. The solvent effects identified in this study, revealed that the most efficient extraction medium for phenolic compounds was methanol for *P. californicum* oak; while acetone extraction of *P. californicum* mesquite showed the lowest content of phenolic compounds. Comparison of solvent effects in characterization of total flavonoid content revealed that aqueous *P. californicum* oak was the most efficient extraction medium. The acetone extract of trunk samples revealed that the highest radical scavenging activity and ferric-reducing antioxidant power activity and the highest superoxide scavenging activity was obtained in acetone extracts of *Bucida buceras* leaves. The various extracts of *Bucida buceras* exhibited potent scavenging activity and indeed, were superior to commercial standards such as ascorbic acid or the flavonoid rutin.

Many plants contain phytochemicals that are beneficial for general health. Investigations such as the present study are progressively characterizing bioactivities of plant products and expanding their use in healthcare.
